# Yield of soybean genotypes identified through GGE biplot and path analysis

**DOI:** 10.1371/journal.pone.0274726

**Published:** 2022-10-12

**Authors:** Welder José dos Santos Silva, Francisco de Alcântara Neto, Wahidah H. Al-Qahtani, Mohammad K. Okla, Abdulrahman Al-Hashimi, Paulo Fernando de Melo Jorge Vieira, Geraldo de Amaral Gravina, Alan Mario Zuffo, Alexson Filgueiras Dutra, Leonardo Castelo Branco Carvalho, Ricardo Silva de Sousa, Arthur Prudêncio de Araujo Pereira, Wallace de Sousa Leite, Gabriel Barbosa da Silva Júnior, Adriana Conceição da Silva, Marcos Renan Lima Leite, Renato Lustosa Sobrinho, Hamada AbdElgawad

**Affiliations:** 1 Plant Science Department, Federal University of Piauí, Teresina, Piauí, Brazil; 2 Department of Food Sciences & Nutrition, College of Food and Agricultural Sciences, King Saud University, Riyadh, Saudi Arabia; 3 Botany and Microbiology Department, College of Science, King Saud University, Riyadh, Saudi Arabia; 4 Brazilian Agricultural Research Corporation, Teresina, Piauí, Brazil; 5 State University of the North Fluminense Darcy Ribeiro, Rio de Janeiro, Brazil; 6 State University of Maranhão, Balsas, Maranhão, Brazil; 7 Federal Rural University of the Amazônia, Belém, Pará, Brazil; 8 Department of Agricultural and Soil Engineering, Federal University of Piauí, Teresina, Piauí, Brazil; 9 Federal University of Ceará, Fortaleza, Ceará, Brazil; 10 Department of Agronomy, Federal University of Technology, Pato Branco, Paraná, Brazil; 11 Department of Biology, University of Antwerp, Antwerpen, Belgium; KGUT: Graduate University of Advanced Technology, ISLAMIC REPUBLIC OF IRAN

## Abstract

Genotype × environment (G×E) interaction is an important source of variation in soybean yield, which can significantly influence selection in breeding programs. This study aimed to select superior soybean genotypes for performance and yield stability, from data from multi-environment trials (METs), through GGE biplot analysis that combines the main effects of the genotype (G) plus the genotype-by-environment (G×E) interaction. As well as, through path analysis, determine the direct and indirect influences of yield components on soybean grain yield, as a genotype selection strategy. Eight soybean genotypes from the breeding program of Empresa Brasileira de Pesquisa Agropecuária (EMBRAPA) were evaluated in field trials using a randomized block experimental design, in an 8 x 8 factorial scheme with four replications in eight different environments of the Cerrado of Northeastern Brazil during two crop seasons. Phenotypic performance data were measured for the number of days to flowering (NDF), height of first pod insertion (HPI), final plant height (FPH), number of days to maturity (NDM), mass of 100 grains (M100) and grain yield (GY). The results revealed that the variance due to genotype, environment, and G×E interaction was highly significant (P < 0.001) for all traits. The ST820RR, BRS 333RR, BRS SambaíbaRR, M9144RR and M9056RR genotypes exhibited the greatest GY stability in the environments studied. However, only the BRS 333RR genotype, followed by the M9144RR, was able to combine good productive performance with high yield stability. The study also revealed that the HPI and the NDM are traits that should be prioritized in the selection of soybean genotypes due to the direct and indirect effects on the GY.

## Introduction

The tropical savannas (Cerrado) of the states of Maranhão and Piauí form one of the last agricultural frontiers in Brazil, whose expansion of soybean (*Glycine max* (L.) Merrill) cultivation areas was due to advances in plant breeding [1,2]. Despite this context, considering the large extension of the Cerrado areas associated with the constant annual variations of climatic elements (rainfall, temperature and relative air humidity), the search for genotypes with high performance and yield stability is essential for the producer market.

The phenotypic expression results from the G×E interaction that can significantly influence the performance of genotypes in multi-environment trials (METs), justifying the need to examine whether the response of this interaction is promoted by the difference in variability between the genotypes in the environments (simple interaction) or if the superiority of the genotypes shows inconsistency in relation to environmental variation (complex interaction) [3,4]. In particular, the stability of a genotype reveals its homogeneous performance in different environments. In contrast, adaptability reveals a genotype’s preferred target environments to achieve high yields [5].

Several statistical methods described in the literature have been used to analyze the G×E interaction and the adaptability and stability of genotypes tested in different environments [6–10]. The biplot GGE analysis developed by Yan et al [11,12] is an effective multivariate method widely applied in METs of different agricultural crops, grouping the genotype effect (G) with the multiplicative effect of the G x E interaction and submitting them principal component (PC) analysis that results in different graphic configurations (biplots) that provide information about the performance and yield of the genotype, as well as identifying mega-environment formation and ranking superior and stable genotypes and specific genotype combinations with environments [13–15].

A concomitant alternative to the GGE biplot analysis is to examine the yield components that are closely correlated with yield, since identifying the existence and degree of this correlation allows the breeder to make simultaneous changes in yield by improving other specific associated characteristics. However, estimates through simple correlations are limited by not being able to determine the relative importance of the direct and indirect influences of the characters that make up the grain yield [16–19].

The path analysis developed by Wright [20] is a method used in breeding programs to unfold the estimated correlations in direct and indirect effects of characters associated with a main variable (dependent variable) [21].

Thus, considering the strong influence of the environment on the phenotype of plants, this study aimed to select soybean genotypes for performance and yield stability accessed by GGE biplot analysis. Furthermore, based on the environments identified by the GGE biplot graphical tool, to verify using the path analysis the relative contribution (direct and indirect effects) of yield components on GY as a breeding strategy.

## Materials and methods

### Genetic material

The genetic material used in this study belongs to the maturity groups (MG) recommended for low latitude regions [22], developed by the EMBRAPA soybean breeding program, characterized by soybean genotypes (M9144RR, M8766RR, M9056RR, ST820RR, BRS SambaíbaRR, BRS 333RR, BRS 9090RR, and BRS 8990RR) of determined growth and MG ranging from 8.2 to 9.4.

### Experimental design and conditions

Yield trials were carried out in the field in eight different environments of the Cerrado of Northeast Brazil, of which five of them (Tasso Fragoso–MA, São Raimundo da Mangabeiras–MA, Uruçuí –PI, Baixa Grande do Ribeiro–PI, and Balsas–MA) in the 2013/2014 crop season and three other environments (Chapadinha–MA, São Raimundo das Mangabeiras–MA, and Tasso Fragoso–MA) in the 2014/15 crop season (Table 1).

**Table 1:** Geographic coordinates, sowing dates, climatic variables (rainfall, temperature, and relative humidity) of the municipalities where the experiments were installed in the 2013/14 and 2014/15 crop seasons.

Crop seasons	E	Sowing date	Rainfall (mm)	Temp. (°C)	Rh (%)	Alt. (m)	Latitude	Longitude
**2013/14**	E_1_	26/11/2013	687	27.5	86.1	242	08°28’30” S	45°44’34” W
	E_2_	18/12/2013	607	27.5	85.8	234	07°01’09” S	45°28’51” W
	E_3_	12/12/2013	618	27.5	86.3	167	07°13’46” S	44°33’22” W
	E_4_	11/12/2013	618	27.5	86.4	325	07°51’01” S	45°12’49” W
	E_5_	28/11/2013	689	27.5	85.9	247	07°31’57” S	46°02’08” W
**2014/15**	E_6_	05/02/2014	1120	27.6	83.4	105	03°44’30” S	43°21’37” W
	E_7_	15/12/2014	655	27.4	79.3	234	07°01’09” S	45°28’51” W
	E_8_	25/11/2014	661	27.4	78.8	242	08°28’30” S	45°44’34” W

E = Environment; Alt. = Altitude; Temp. = Temperature average; Rh = Relative humidity; E_1_ = Tasso Fragoso (MA), E_2_ = São Raimundo das Mangabeiras (MA), E_3_ = Uruçuí (PI), E_4_ = Baixa Grande do Ribeiro (PI), E_5_ = Balsas (MA), E_6_ = Chapadinha (MA), E_7_ = São Raimundo das Mangabeiras (MA), E_8_ = Tasso Fragoso (MA). Source: INMET.

The experimental design followed a randomized block design, adopting an 8 x 8 factorial scheme with four replications. The experimental plot was composed of four 5-meter rows spaced at 0.5 m between rows. The two center rows were used for data collection (useful area), disregarding the 0.5 m from the ends as a border area. All the trials were sown manually, uniformly distributing 15 seeds per meter (300,000 plants ha^-1^). The seeds were previously treated with fipronil, pyraclostrobin, and thiophanate-methil at a dosage of 2.0 mL kg^-1^ and, just before sowing, inoculated with *Bradyrhizobium japonicum* using a Cell tech® liquid inoculant (Monsanto, USA) at a dosage of 75 mL/27 kg of seeds. Fertilization was carried out with the application, on average, of 60 kg ha^-1^ P_2_O_5_ and 80 kg ha^-1^ of K_2_O, distributing 40 kg ha^-1^ K_2_O before sowing and 40 kg ha^-1^ K_2_O applied 20 days after emergence (DAE). Pre-emergent and post-emergent herbicides were used at all trial sites. The fungicide applications were performed with difeconazole, with the first application at 25 DAE, and the others at 15-day intervals after the last application.

### Evaluations of yield components

All phenotypic data were collected from five randomly selected and tagged plants in each plot. NDF were recorded as the period between sowing and the start of flowering, when 50% of the flowers are open (stage R1) to R2 (full flowering). HPI was measured with a graduated ruler, from the soil to the insertion point of the 1st pod. FPH was measured from the base of the plant at full maturity stage to the upper end of the main stem. NDM were recorded as the period between sowing to full maturity (95% of the pods have reached their full mature color).

All of the trials were harvested manually. The GY and M100 were determined in the useful area of each plot (4 m^2^), five days after the plants reached full maturation. The pods were manually threshed and the harvested grains were stored in properly identified paper bags for later determination of the grain mass per useful area of each plot. The GY (kg ha^-1^) was estimated with the humidity adjustment to 13% as determined by the drying oven method at 105°C for 24 h [23]. The M100 was determined by separating the grains according to the method established by Brasil [23], and the masses quantified using a precision balance.

### Data analysis

The phenotypic data were collected in the eight environments, the homogeneity of variance was verified by the Bartlett [24] test and, given the assumption of homogeneity, the data were submitted to joint analysis of variance (ANOVA) to determine the significance level of genotypes (G), environments (E) (represented by crop seasons and location), and G x E interaction using fixed linear model in R software [25].

To explain the G×E interaction, the multivariate stability analysis was performed graphically based on the GGE biplot model [26] using R package GGEBiplotGUI. Singular value decomposition (SVD) of the first two principal components was used to fit the GGE biplot model according to the equation below:

$$GGE=\sum_{K=1}^{n}\lambda_{k}\gamma_{ik}\alpha_{jk}+\rho_{ij}$$

where GGE is the matrix of the effects of genotypes added to the effects of the interaction; λ_k_ is the k^-th^ singular value of the original matrix interactions (GE); γ_ik_ is the element corresponding to the i^-th^ genotype in the k^-th^ singular vector of the GE matrix column; α_jk_ is the element corresponding to the j^-th^ environment in the k^-th^ singular vector of the GE matrix row; and ρij is the residual related to the adjustment.

GGE biplots are graphical tools to demonstrate the G×E interaction and the classification of genotypes based on the mean and stability obtained for an evaluated character. The generated graph is based on METs evaluation for environmental stratification (who-won-where pattern), genotype evaluation (mean versus stability) and tested environment ranking (discriminatory versus representative). The classification of genotypes was established in ascending order of each stability parameter. The biplots were based on partitioning with singular value = 2, transformed (transform = 0), centered on the environment (centering = 2) and standardized with standard deviation (scaling = 0).

Estimates of phenotypic correlation coefficients between traits were obtained following the methodology described by Steel and Torrie [27]. The diagnosis of multicollinearity was performed for all environments, based on the Variance Inflation Factor (VIF) obtained according to equation below:

$${VIF}_{i}=\frac{1}{\left(1-R_{i}^{2}\right)}$$

where R_i_^2^ is the multiple determination coefficient for the linear regression of Xi on the other covariates. The VIF estimates how much the variance of a regression coefficient is inflated due to multicollinearity presented in the model [28]. The correlations were divided into direct and indirect effects among the studied variables relative to soybean GY through the path analysis proposed by Wright [20].

## Results and discussion

The analysis of variance showed that soybean GY, NDF, HPI, FPH, NDM, and M100 was significantly (p < 0.001) affected by environments main effects, genotypes main effects and G x E interaction effects (Table 2), demonstrating that differences between environments, represented by crop seasons and locations, require prudence in the selection process. Thus, due to the lack of consistency in the performance of the genotypes, we cannot recommend them for all environments in our study. According to Olanrewaju et al. [29], progress in selection requires considering the influence that the environment has on genotypes, mainly in the breeding of polygenic traits that are highly influenced by the environment. Furthermore, it is reported by Bhartiya et al. [30] that the selection of genotypes should prioritize, in addition to the G x E interaction, the number of years or harvests over the number of locations, considering that environmental heterogeneity over the years tends to increase with constant climate changes in regions and areas of cultivation.

**Table 2:** Summary of the joint analysis of variance for number of days to flowering (NDF), height of first pod insertion (HPI), final plant height (FPH), number of days to maturity (NDM), mass of 100 grains (M100), and grain yield (GY).

SV	DF	Mean squares					
		NDF	HPI	FPH	NDM	M100	GY
**G**	7	143.99***	43.28***	1,027.5***	429.16***	1,624.2***	1,146,838.0***
**E**	7	767.29***	368.39***	4,014.1***	1,184.93***	4,505.0***	10,749,342.0***
**Block / E**	24	2.68**	11.84*	143.2**	3.37^ns^	77.2^ns^	302,583.0^ns^
**(G x E)**	49	14.59***	14.99***	221.1***	35.72***	326.2***	528,336.0***
**Error**	168	1.25	7.36	63.70	3.64	94.70	268,758.0
**CV (%)**		2.32	18.18	12.43	1.65	6.99	15.54

*** = Significant at 0.001 significance level.

** = Significant at 0.01 significance level.

* = Significant at 0.05 significance level.

ns = non-significant by the F-test (p > 0.05).

G = genotypes; E = environments; SV = Sources of variation; DF = degrees of freedom; CV = coefficient of variation.

**Fig 1:**
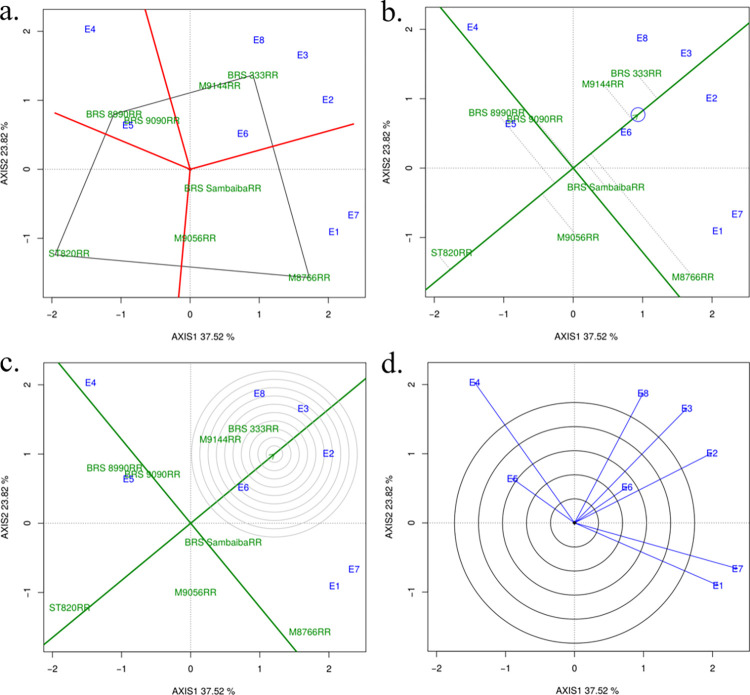
(a) GGE biplot representing the which-won-where, where the genotypes at the vertices of the polygon represent the genotypes indicated for the respective mega-environments formed (red lines). (b) GGE-biplot analysis for “mean performance versus stability” for yield of soybean genotypes. (c) GGE biplot comparing eight genotypes evaluated according to the estimate of an ideal genotype. (d) GGE biplot comparing eight soybean genotypes evaluated according to the discrimination and representativeness of environments for grain yield (kg ha^-1^).

Considering the GY, it is observed that the environments account for the greatest variation in relation to G x E interactions, while the genotypes show the smallest variation. The magnitude of the mean square of the G x E interaction of GY, resulting from the product of the mean square with the degree of freedom, was about 3.23 times greater than the magnitude of the genotypes, reinforcing the significant differences in the genotypic response in the MET of this study. The GGE biplot based on this data set is shown in Fig 1, with the scores of PC1 on the x-axis and the scores of PC2 on the y-axis, for genotypes and environments.

### GGE biplot analysis

GGE biplot aided on finding the best-performing genotypes adapted at the specific location or stable genotype for multiple locations and even determines the most representative locations (mega-environment—ME) for a genotype [13]. In the decomposition applied to the effects of genotype and G x E interaction, considering the first two principal components (PC), the biplot explained 61.34% of the total variation observed, with 37.52% explained by the first principal component (axis 1), and 23.82% by the second principal component (axis 2).

The "which-won-where" graph (Fig 1A) allows for visual grouping of test environments based on G x E crossing between the best genotypes. The genotype at the vertex of the polygon performs best in the environment falling within the sectors. The vertices of the polygon are formed by the genotypes: BRS 333RR, BRS 8990RR, ST820RR and M8766RR. The eight environments were cut into 3 groups by the lines (red) that came out of the origin of the biplot, the groups are formed by (ME1) E4 and E5 (ME2) E1 and E7; (ME3) E2, E3, E6 and E8. The BRS 333RR genotype had the best performance in ME3; the BRS 8990RR had the best performance in ME1; and M8766RR was considered with the best performance in ME2, so these are the most adapted genotypes in these environments. The ST820RR genotype is present in a sector that does not contain environments, meaning that this genotype is not productive in any environment, that is, this genotype is the worst genotype in relation to GY in some or all environments.

The GGE biplot "Mean versus Stability" is an effective tool for the evaluation of genotypes in both aspects [11]. In Fig 1B, the genotypes are arranged along the "average environment-axis" (or AEA) based on their average performance in all environments, with the arrow pointing to the highest yield value. The perpendicular line separates genotypes that produced below average from those that produced above average. Thus, genotypes can be ranked according to their average GY as follows: BRS 333RR > M9144RR > M8766RR > BRS SambaibaRR > BRS 9090RR > general average (GY) > BRS 8990RR > M9056RR > ST820RR.

The length of the projections of each genotype to the AEA (dotted lines) approximates their contributions to the GxE interaction, which is the measure of their instability [11,13]. Therefore, the larger this vector, the lower the yield stability of the genotype throughout the test environments. According to Fig 1B, BRS 333RR and M9144RR, are located further upstream to the average yield value and with relatively low projections in relation to the AEA, being the genotypes that best allied high yield and stability, with their rankings consistent across locations. BRS 333RR and M9144RR are good candidates in breeding programs aimed at selecting broad yield adaptability in soybeans.

The good performance of these genotypes is confirmed when they are compared to a hypothetical ideal genotype represented by the concentric area of Fig 1C, located in the region corresponding to the smallest circle on the AEA [13]. Thus, the BRS 333RR genotype, followed by M9144RR, can be considered the closest to an ideal genotype, that is, able to combine good grain yield performance with high stability throughout the multi-environment productivity trials.

The goal of the test environment assessment is to identify environments that can be used to effectively select superior genotypes for a mega-environment [13]. In this way, the relationship between environments can be seen in the GGE biplot (Fig 1D) designed for this purpose. The test environments with longer vectors are more discriminating with respect to genotypes. Those environments with a short vector are less discriminating, which means that all genotypes tend to perform similarly and little or no information about genotypic differences can be revealed in such an environment, and therefore should not be used as a test environment. The environments (E5 and E6) showed short vectors, meaning they are less discriminating with respect to genotypes. A second utility of Fig 1D is to indicate representative test environments. The test environments that have small angles with AEA (average environment-axis), for example, E2, E3 and E8, are more representative than those that have larger angles, for example, E1, E4 and E7. Therefore, the test environments that are discriminatory and representative (for example, E2, E3 and E8) are ideal test environments for the selection of adapted genotypes. The test environments that are discriminatory but not representative (for example, E1, E4 and E7) are useful for the selection of genotypes adapted specifically in mega-environments; or for the selection of unstable genotypes if the test environment is a single mega-environment [11].

### Path analysis

Understanding the relationship of the various yield components with grain yield can be explored for use as an indirect selection criterion to improve genotype yield in breeding programs [31]. In this study, it was verified that the phenotypic and genotypic correlations are similar, indicating that there is an intrinsic association between several characters.

**Fig 2:**
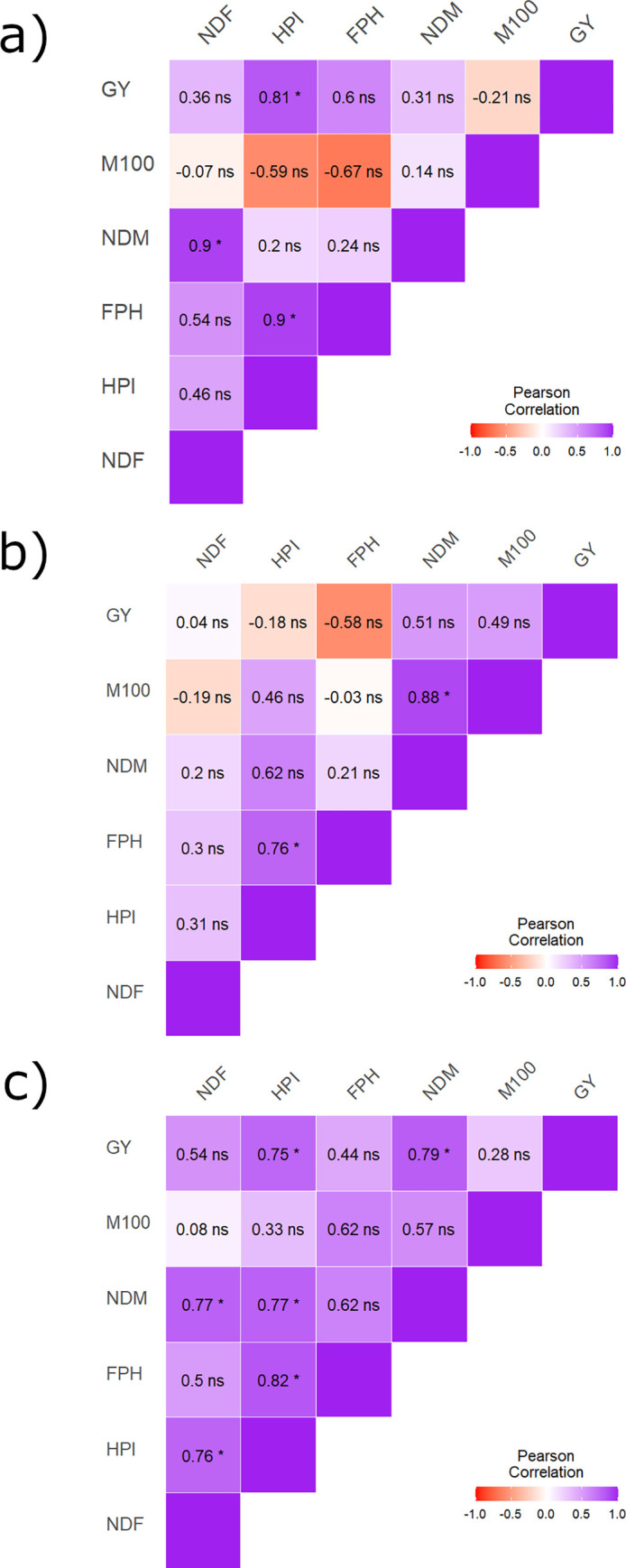
Estimates of the phenotypic correlation between the variables NDF, HPI, FPH, NDM, M100 and GY in tree mega-environments ((a) ME1, (b) ME2, and (c) ME3) used to test soybean genotypes.

The correlation analysis (Fig 2) showed different degrees of association between GY and the yield components in each mega-environment. There was a positive and significant association (*p* < 0.05) of GY with HPI in the ME1; and with HPI and NDM in the ME3. Similarly, a positive and significant correlation (*p* < 0.05) was observed in HPI with FPH, and NDM with NDF (ME1); HPI with FPH, and NDM with M100 (ME2); and HPI with NDM, FPH and NDF, and NDF with NDM (ME3). No significant correlation was observed between GY and the yield components in ME2. These results are similar to those found by Nogueira et al. [32] and Ferrari et al. [33], who observed that the correlation magnitudes between the same traits had a high oscillation according to the crop seasons and sowing period. The correlation results in each mega-environment indicated the existence of variability among the soybean genotypes with different performances according to the cultivation environment.

Therefore, observing trait correlations relative to the growth environment suggests that selecting soybean genotypes based on yield traits could be efficient when performed in the environment to which the genotype is most adapted [32]. Thus, we can infer a potential indirect selection for GY by the evaluation of HPI in ME1, and HPI and NDM in mega-environment 3. According to Nogueira et al. [32], the interpretation of secondary components effects on the primary must be conducted carefully because when the coefficients of determination are low, the corresponding residual effects are high with fewer significant variables.

The identified strong correlations could be related to factorial linkages through pleiotropy, in which a gene can influence the expression of more than one trait, enabling the simultaneous selection of two or more traits, although selecting only one trait [34]. Furthermore, the correlation coefficient only measured the linear relationships, while nonlinear variables could still be valid. A high correlation does not imply a direct cause-and-effect relationship between the analyzed variables [35]. According to the results obtained in the GGE Biplot analysis the direct effect magnitudes can change based on the growth environment.

**Fig 3:**
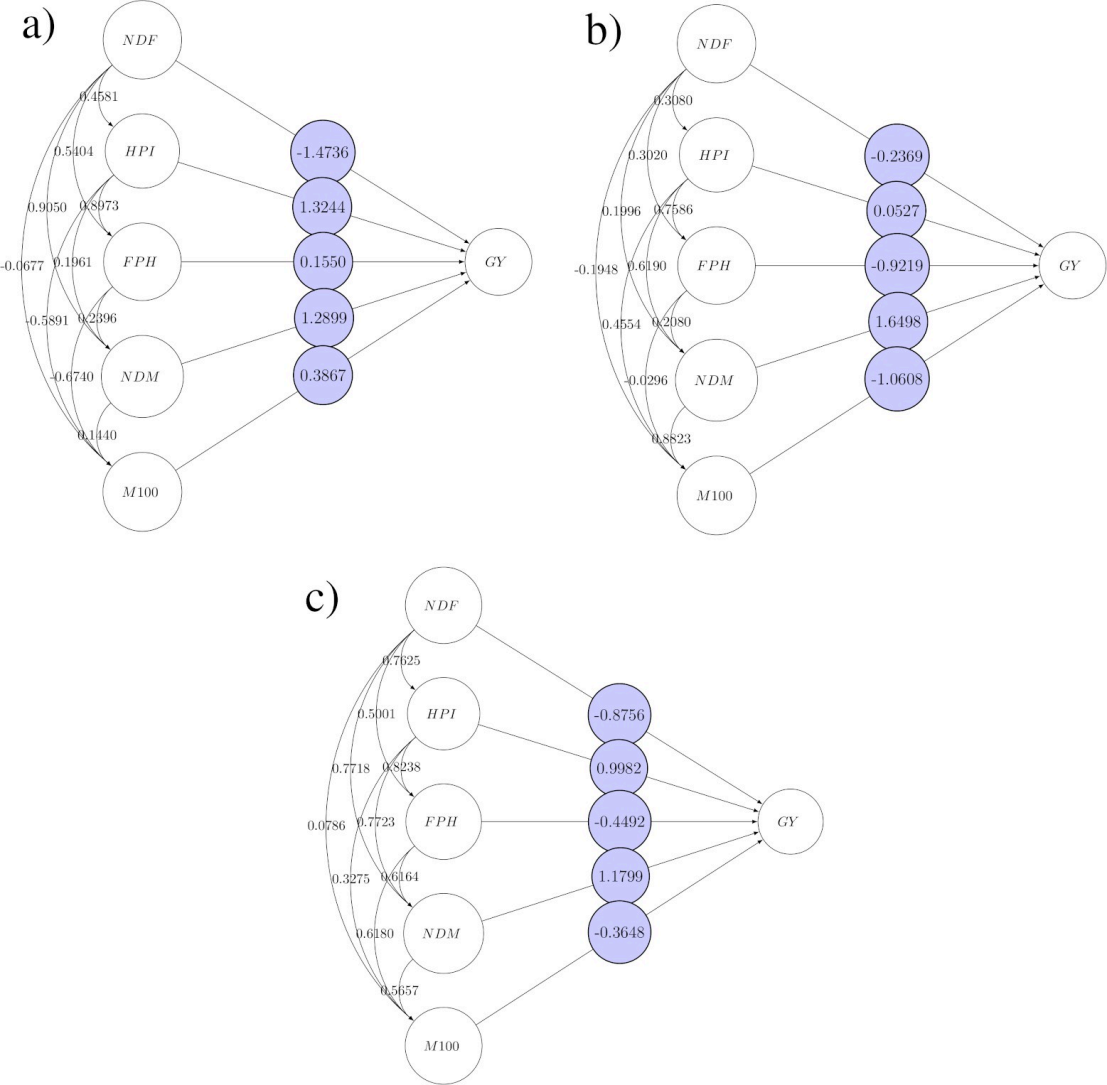
Diagram of path coefficients between the variables NDF, HPI, FPH, NDM, M100 and GY in tree mega-environments ((a) ME1, (b) ME2, and (c) ME3) used to test soybean genotypes.

Justified by the similarity of phenotypic and genotypic correlations, it was prioritized for path analysis, the use of phenotypic data and, proceed with the analyzes within each mega-environment safeguarding the results obtained by GGE analysis. In Mega-environment 1 (Fig 3A) HPI had the greatest direct effect on soybean GY, with direct effect of 1.3244, followed for NDM (1.2899). For ME2 (Fig 3B), NDM was the most important trait for selection of GY (1.6498), and in ME3 (Fig 3C), NDM was the trait that most contributed for GY, followed for HPI (1.1799, and 0.9982, respectively). Thus, the NDM can be considered a key trait, with the greatest contribution, in all environments.

Therefore, for recommendation of new cultivars in the evaluated environments, NDM and HPI, can be used to develop an ideally effective selection index for soybean breeding programs. Similar results were found by Val et al. [36], who found a greater influence of NDM on soybean GY. In a study reported by Zuffo et al. [37], also was observed the direct effects of HPI on soybean GY.

However, our results do not coincide with those found by Nogueira et al. [32], who found a greater direct phenotypic effect on GY for the number of pods per plant, M100, and number of grains per pod. Alcântara Neto et al. [16], observed a low correlation between HPI and FPH, number of nodes per plant and the average number of pods per plant. Furthermore, they verified the smallest direct and indirect effects on the production of dry matter, M100, and total GY per plant.

HPI showed a low cause-and-effect relationship in the variables studied by Zuffo et al. [38], and for Baig et al. [39], M100 and NDM, showed a strong association with GY as well as direct and indirect positive effects for one or more characters. They also verified that direct selection for these traits would increase the efficiency of genetic gains for GY in soybean.

In this study, NDF, in general, was the variable with the highest negative influence on soybean yield, with negative estimates in terms of direct effects in all mega-environments (-1.4736, -0.2369, and -0.8756, respectively). Considering that genotypes with very different cycles interfere in the consistency of interactions, it is important to evaluate the effect only of the best genotypes with similar flowering cycles.

The current study showed a significant influence of vegetative components in soybean GY. These variables have direct and indirect effects on the yield and its contributing traits. Furthermore, the traits of HPI and NDM must be prioritized in the selection of superior soybean genotypes due to their direct and indirect effects on GY, which were very influential in all cultivation environments. Hence, indirect selection to increase GY is an efficient selection strategy to optimize the genetic gain of soybeans [40], even when considering contrasting environments.

## Conclusions

This study pointed out the effect of the G×E interaction through the GGE biplot analysis, demonstrating the stability of the genotypes and the representativeness and discriminative capacity of the environments for grain yield. The GGE biplot analysis made it possible to identify stable and high-yield genotypes in the environments. The genotypes ST820RR, BRS 333RR, BRS SambaíbaRR, M9144RR and M9056RR are, in this sequence, the genotypes the showed the greatest stability of grain yield in the average of the environments studied. However, only the BRS 333RR genotype, followed by the M9144RR, was able to combine good productive performance with high productive stability throughout the multi-environment productivity trials. Thus, according to the growing conditions in each mega-environment, the components yield present different behavior and, therefore, such information can be useful in the soybean breeding process using the genotypes of this study.

This study highlighted the importance of evaluating vegetative components in multi-environment trials that aims to select genotypes with higher grain yield in soybean breeding programs. Vegetative traits can also be considered as key in the development of early selection indices when the objective is to obtain genotypes with good stability and adaptability in this crop.
