# Correction: Yield of soybean genotypes identified through GGE biplot and path analysis

**DOI:** 10.1371/journal.pone.0320098

**Published:** 2025-03-05

**Authors:** Welder José dos Santos Silva, Francisco de Alcântara Neto, Wahidah H. Al-Qahtani, Mohammad K. Okla, Abdulrahman Al-Hashimi, Paulo Fernando de Melo Jorge Vieira, Geraldo de Amaral Gravina, Alan Mario Zuffo, Alexson Filgueiras Dutra, Leonardo Castelo Branco Carvalho, Ricardo Silva de Sousa, Arthur Prudêncio de Araujo Pereira, Wallace de Sousa Leite, Gabriel Barbosa da Silva Júnior, Adriana Conceição da Silva, Marcos Renan Lima Leite, Renato Lustosa Sobrinho, Hamada AbdElgawad

There are errors in the Funding statement. The correct Funding statement is as follows: This study was financed in part by King Saud University, Ri-yadh, Saudi Arabia under Researchers Support Project number (RSP-2021/219) awarded to MKO, AAH, and HA, and by the Coordenação de Aperfeiçoamento de Pessoal de Nível Superior – Brasil (CAPES) – Finance Code 001 with one awarded for author WJSS. The funders had a role in study design, data collection and analysis, decision to publish, or preparation of the manuscript.

The Acknowledgement section is not indicated. The correct Acknowledgement section is as follows: The authors extend their appreciation to the researchers supporting project number (RSP-2021/219), King Saud University, Riyadh, Saudi Arabia.
